# Prevalence and Risk Factors of Zoonotic Dermatophyte Infection in Pet Rabbits in Northern Taiwan

**DOI:** 10.3390/jof8060627

**Published:** 2022-06-13

**Authors:** Che-Cheng Chang, Wittawat Wechtaisong, Shih-Yu Chen, Ming-Chu Cheng, Cheng-Shu Chung, Lee-Shuan Lin, Yi-Yang Lien, Yi-Lun Tsai

**Affiliations:** 1 Department of Veterinary Medicine, College of Veterinary Medicine, National Pingtung University of Science and Technology, Pingtung 912, Taiwan; goodteam3373@yahoo.com.tw (C.-C.C.); wittwech@gmail.com (W.W.); msychen@ucdavis.edu (S.-Y.C.); mccheng@mail.npust.edu.tw (M.-C.C.); cschung@mail.npust.edu.tw (C.-S.C.); lslin@mail.npust.edu.tw (L.-S.L.); yylien@mail.npust.edu.tw (Y.-Y.L.); 2 Guting Veterinary Hospital, Taipei 100, Taiwan; 3 Department of Population Health and Reproduction, School of Veterinary Medicine, University of California, Davis, CA 95616, USA; 4 Research Center of Animal Biologics, National Pingtung University of Science and Technology, Pingtung 912, Taiwan; 5 Veterinary Medical Teaching Hospital, National Pingtung University of Science and Technology, Pingtung 912, Taiwan

**Keywords:** dermatophytes, *Trichophyton mentagrophytes* complex, *Microsporum canis*, risk factors, pet rabbits, Taiwan

## Abstract

Dermatophytes are the group of keratinophilic fungi that cause superficial cutaneous infection, which traditionally belong to the genera *Trichophyton*, *Microsporum*, and *Epidermophyton*. Dermatophyte infection is not only a threat to the health of small animals, but also an important zoonotic and public health issue because of the potential transmission from animals to humans. Rabbit dermatophytosis is often clinically identified; however, limited information was found in Asia. The aims of this study are to investigate the prevalence and to evaluate the risk factors of dermatophytosis in pet rabbits in Northern Taiwan. Between March 2016 and October 2018, dander samples of pet rabbits were collected for fungal infection examination by Wood’s lamp, microscopic examination (KOH preparation), fungal culture, and PCR assay (molecular identification). Z test and Fisher’s exact test were performed to evaluate the potential risk factors, and logistic regression analysis was then performed to build the model of risk factors related to dermatophyte infection. Of the collected 250 dander samples of pet rabbits, 29 (11.6%) samples were positive for dermatophytes by molecular identification. In those samples, 28 samples were identified as the *T. mentagrophytes* complex and 1 sample was identified as *M. canis*. Based on the results of the Firth’s bias reduction logistic analyses, animal source (rabbits purchased from pet shops) and number of rearing rabbits (three rabbits or more) were shown as the main risks for dermatophyte infection in the pet rabbits in Taiwan. The results of the present study elucidate the prevalence of rabbit dermatophyte infection, pathogens, and risk factors in Taiwan, and provide an important reference for the prevention and control of rabbit dermatophytosis.

## 1. Introduction

Dermatophytes, referred to as the ringworm fungi, are traditionally divided into three closely related genera, including *Epidermophyton*, *Trichophyton*, and *Microsporum* spp. [1,2]. *Trichophyton* spp. and *Microsporum* spp. cause skin diseases in animals, such as *T. mentagrophytes*, *T. verrucosum*, and *M. canis*, which are known as zoophilic dermatophytes. The transmission of these zoophilic dermatophytes occurs via infective arthrospores coming from the hair coat of infected animals or the environment [3,4]. Infected animals represent an important role of fungal transmission to humans, which cause tinea corporis, tinea capitis, and tinea barbae in humans [3,4,5,6].

Dermatophytoses are common fungal infections in rabbit farm, laboratory, and pet rabbits [3,7,8,9,10,11,12,13,14]. The most common agents of dermatophytoses in rabbits belong to the *T. mentagrophytes* complex and *M. canis* species [3,4,8,12,15,16,17]. The infections may cause alopecia, redness scaly, and scurf localized mainly on the rabbit face, head, auricles, and dorsal area of the neck [18,19,20]. Several reports have also revealed that rabbits can transmit dermatophytes to humans by direct contact [9,21,22,23]. Therefore, understanding the epidemiology of dermatophytes in rabbits will play an important part on reducing its incidence in both humans and rabbits [24].

In Taiwan, various epidemiological surveys of human dermatophytoses have been reported [25,26,27,28,29,30,31]. *M. ferrugineum* and *T. violaceum* are the main causative agents of human dermatophytoses in Northern and Southern Taiwan, respectively [27,29]. In 2013, Taiwan’s National Health Insurance Research Database showed that tinea pedis, tinea cruris, and onychomycosis were the three most common types of dermatophytoses [26]. Some cases were found to be related to their pet animals, such as rabbits; however, the knowledge of epidemiology and risk factors of rabbit dermatophytoses in Taiwan are limited. This study aims to determine the prevalence and risk factors of dermatophyte infection in pet rabbits in Northern Taiwan, especially in Taipei and New Taipei. The findings can be used to formulate the health and welfare policy and provide an important reference for the prevention and control policy of zoonotic fungal diseases in the country.

## 2. Materials and Methods

### 2.1. Sample Collection

From March 2016 to October 2018, a total of 250 pet rabbits were examined for dermatophyte infection at Guting animal hospital in Taipei City, Rabbit Saving Association, and Taiwan Stray Bunny Protection Association (IACUC Approval No. NPUST-106-007). The pet rabbits’ data, including breed, weight, age, gender, neuter status, source, location, living space, number of rearing rabbits, rearing with other animals, history of ectoparasite infestation, and season of sample collection, were collected for analysis of potential risk factors of dermatophyte infection.

For each pet rabbit, Wood’s lamp was firstly used to detect the fluorescent reaction of the dermatophytes [10,32]. Some hairs were plucked and placed on a glass slide. Dandruff and keratin were dissolved with 10% potassium hydroxide (KOH), and then examined under a microscope for dermatophyte infection [33]. In addition, dander samples collected from each pet rabbit by using the Mackenzie brush technique were applied for dermatophyte culture and molecular identification [34].

### 2.2. Dermatophyte Culture and Morphological Identification

All dander samples were cultured on Mycosel agar plates (Coning Technology, Kaohsiung, Taiwan) and incubated at 25 °C for up to 4 weeks [8]. The fungal growth was checked every 2–3 days during incubation. After fungal colonies were observed, the colonies were sub-cultured with potato dextrose agar plates (BioPioneer Tech Co., Ltd., New Taipei, Taiwan) for purification and storage.

Macroscopic morphology was observed by the size, texture, and color of colonies on agar plates [35]. The microscopic examination was performed by staining the dermatophyte with lactophenol cotton blue, and the fungal characteristics including size, shape, and arrangement of microconidia and macroconidia were observed. The classification of fungal colonies was identified according to the morphological keys [36,37]. When fungal growth was not observed within 4 weeks, the sample was considered as negative on dermatophyte isolation.

### 2.3. Molecular Identification

All DNA samples were extracted from hyphae of the suspected dermatophyte isolates using an MX-16 automatic nucleic acid extractor (Compacbio Sciences, Burlingame, CA, USA) with a Maxwell 16 Tissue DNA Purification kit (PROMEGA Corp., Madison, WI, USA) following the manufacturer’s instructions. All DNA samples were stored at −20 °C until PCR processing for dermatophyte detection.

Dermatophyte DNA was detected by using PCR assay [38]. The primer forward (ITS1) 5′-TCCGTAGGTGAACCTGCGG-3′ and reverse (ITS4) 5′-TCCTCCGCTTATTGATATGC-3′ were used to amplify fragments of the rRNA internal transcribed spacer (*ITS*) region of *Trichophyton* spp. (603 bp.) and *Microsporum* spp. (632 bp.) [38]. PCR mixture was set up as follows: 2.5 μL of DNA template serially diluted with sterile distilled water (original, 10×, 50×, 200×), 0.3125 μL of 10 μM of each primer, 2.5 μL of PCR buffer (Genomics BioSci & Tech Co., Ltd., Taipei, Taiwan), 2 μL of 2.5 mM dNTPs mixture (Genomics BioSci & Tech Co., Ltd., Taipei, Taiwan), 0.75 μL of 50 mM MgCl_2_ (Genomics BioSci & Tech Co., Ltd., Taipei, Taiwan), 0.125 μL of Taq DNA polymerase (Genomics BioSci & Tech Co., Ltd., Taipei, Taiwan), and adjusted to a final volume of 25 μL with sterile distilled water. The PCR condition was described by White et al. [38]. The expected PCR products were separated using a 2% agarose gel stained with nucleic acid stain (Genomics BioSci & Tech Co., Ltd., Taipei, Taiwan), and the results were visualized under UV light using UVIdoc HD5 (Uvitec, Cambridge, UK).

The dermatophyte PCR-positive products from hyphae samples were purified using a Plus DNA Clean/Extraction Kit (GMbiolab Co., Ltd., Taichung, Taiwan) and sent for nucleotide sequencing (Genomics BioSci & Tech Co., Ltd., Taipei, Taiwan). Sequence data were compared with known sequences deposited in the GenBank database using the NCBI nucleotide BLAST tool. For genetic analysis, validated sequences were aligned and analyzed by using MegAlign (DNASTAR, Inc., Madison, WI, USA).

### 2.4. Statistical Analyses

R statistical software (R 3.5.2 R Core Team, 2018) was used in this study to perform descriptive statistical analyses and to assess the association between dermatophyte infection and potential risk factors. The descriptive statistics were presented as mean and standard deviation for measurement data, and medians and interquartile ranges for categorical data. Z test was used for the continuous variable (weight), and Fisher’s exact test was used for the categorical variables (rabbit breed, age, gender, neuter status, source, location, living space, number of rearing rabbits, living with other animals, ectoparasite and season), with significance at *p* < 0.05. Logistic regression analysis was then performed to build the model of risk factors related to dermatophyte infection [39].

The Kappa value coefficient was used to interpret the consistency of the results among the detection methods of Wood’s lamp, microscopic examination, and culture/molecular identification, and Excel software was used for calculation and analysis. According to Fleiss’s Kappa value, five levels of agreement were considered as follows: 0.0–0.20 (extremely low), 0.21–0.40 (fair), 0.41–0.60 (moderate), 0.61–0.80 (substantial), and 0.81–1 (almost perfect) [40,41].

## 3. Results

### 3.1. Demography of Recruited Pet Rabbits

**Figure 1:**
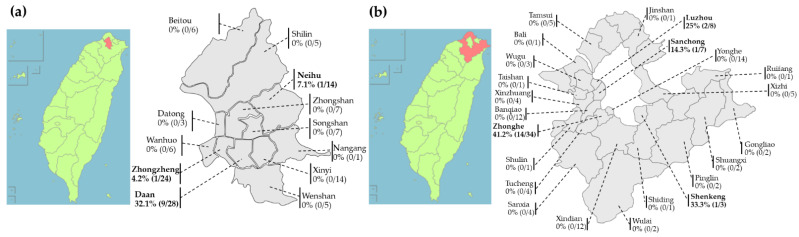
Geographic distribution of pet rabbits recruited from Northern Taiwan. The prevalence of dermatophyte infection in rabbits is higher in (**b**) New Taipei City (13.3%, 18/135) than that in (**a**) Taipei City (9.56%, 11/115), *p* value = 0.4297.

From March 2016 to October 2018, a total of 250 pet rabbits were examined at Guting animal hospital, Taiwan Rabbit Saving Association, and Taiwan Stray Bunny Protection Association. The average weight of the recruited rabbits was 1.64 ± 0.76 kg, and the average age was 34.88 ± 30.60 months. Among the animals, 135 (54%) rabbits were from districts of New Taipei City, while 115 (46%) rabbits were from districts of Taipei City (Figure 1). The pet rabbits were identified into 14 breeds, including 113 (45.2%) mixed breed, 50 (20%) dodge rabbits, 39 (15.6%) Dutch dwarf rabbits, 28 (11.2%) lion head rabbits, 4 (1.6%) Polish white rabbits, 3 (1.2%) miniature rabbits, 3 (1.2%) English spotted rabbits, 2 (0.8%) dwarf Hotot rabbits, 2 (0.8%) New Zealand white rabbits, 2 (0.8%) Himalayan rabbits, 1 (0.4%) standard chinchilla rabbit, 1 (0.4%) Havana rabbit, 1 (0.4%) American fuzzy lop rabbit, and 1 (0.4%) crème d’Argent rabbit. Except “location”, the numbers of pet rabbits in the other ten categorical variables, including breed, age, gender, neuter status, source, living space, number of rearing rabbits, rearing with other animals, ectoparasite infestation, and season of sample collection, were recorded and shown in Table 1.

### 3.2. Fungal Detection and Identification

**Figure 2:**
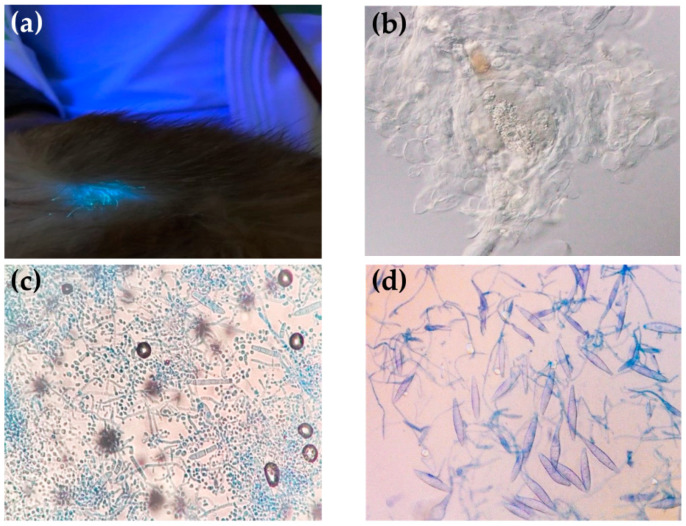
Dermatophyte detection methods used in this study: (**a**) fluorescent reaction on the rabbit body examined by Wood’s lamp; (**b**) presence of fungal spore with a 10% KOH preparation; (**c**) microscopic characteristics of *Trichophyton* spp.; (**d**) microscopic characteristics of *Microsporum* spp.

Out of 250, 8 (3.2%) pet rabbits presented fluorescent reaction on the bodies examined by Wood’s lamp (Figure 2a). All of the dander samples were tested with a 10% KOH preparation, of which nine (3.6%) samples showed the presence of fungal spores (Figure 2b). Based on culture, 29 (11.6%) samples were suspected of growth of dermatophytes. In those suspected samples, 28 samples were identified as *Trichophyton* spp. (Figure 2c), and 1 sample was identified as *Microsporum* spp. (Figure 2d), by colony morphology and microscopic examination, respectively.

The PCR results revealed that all 29 suspected colonies harbored dermatophyte DNA fragments, which were 28 *Trichophyton* and 1 *Microsporum* PCR-positive samples. From *Trichophyton* PCR-positive products, 11 samples were randomly selected and sent for sequencing. Ten sequences showed 100% similarity with *T. interdigitale* isolates (KY930378, MN886818, and MN691064), and one sequence showed 100% similarity with the *T. benhamiae* isolate (KY827233) listed in the GenBank database. Based on the phylogeny, *T. interdigitale* and *T. benhamiae* were assumed as the *T. mentagrophytes* complex. The sequence from the *Microsporum* PCR-positive sample showed 100% similarity with the *M. canis* (MN808763) listed in the GenBank database.

In the comparison of positive results among different detection methods, Wood’s lamp examination, microscopic examination (KOH preparation), and molecular identification was shown as follows: (1) In the 250 recruited pet rabbits, 29 rabbits were detected positive for dermatophytes by molecular identification, while 8 rabbits were positive by Wood’s lamp examination. Four rabbits were detected positive by both methods; (2) In the 250 recruited pet rabbits, 29 rabbits were positive for dermatophytes by molecular identification, while 9 rabbits were positive by microscopic examination (KOH preparation). Five rabbits were detected positive by both methods; (3) In the 250 recruited pet rabbits, 8 rabbits were positive for dermatophytes by Wood’s lamp examination, while 9 rabbits were positive by microscopic examination (KOH preparation). Four rabbits were detected positive by both methods.

Comparative analysis of the methods for dermatophyte detection was performed by using the Kappa value coefficient. The Kappa value of the dermatophyte detection between Wood’s lamp examination and molecular identification was 0.17, indicating that the detection results of the two methods were extremely low consistency. The Kappa value of the dermatophyte detection between microscopic examination (KOH preparation) and molecular identification was 0.22, indicating that the detection results of the two methods were in very low consistency. In addition, the Kappa value of the dermatophyte detection between Wood’s lamp and microscopic examination (KOH preparation) was 0.42, indicating that the detection results of the two methods were in moderate consistency.

### 3.3. Prevalence and Potential Risk Factors of Rabbit Dermatophytoses

In this study, according to the results of molecular identification, the prevalence of dermatophyte infection in pet rabbits was 13.9% (29/250). The categorical variables were analyzed using Fisher’s exact test with significance at *p* < 0.05, and the prevalence of dermatophyte infection in each categorical variable is shown in Table 1. The variables, including source, number of rearing rabbits, rearing rabbits with other animals, and ectoparasite infestation, were related to significant differences in the prevalence and considered as the potential risk factors. In the northern cities of Taiwan, the prevalence of dermatophyte infection was higher in New Taipei City (13.3%, 18/135) than that in Taipei City (9.56%, 11/115) (*p* value = 0.4297). In Taipei City, eleven samples from Daan District (9/28, 32.1%), Neihu District (1/14, 7.1%), and Zhongzheng District (1/24, 4.2%) were detected as dermatophyte positive, but those from the rest of the districts were all negative (Figure 1a). In New Taipei City, 18 samples from Zhonghe District (14/34, 41.2%), Shenkeng District (1/3, 33.3%), Luzhou District (2/8, 25%), and Sanchong District (1/7, 14.3%) were detected as dermatophyte positive, but those from the rest of the districts were all negative (Figure 1b). Regarding the source of pet rabbits, the prevalence of dermatophyte infection in rabbits bought from pet shops (18.3%) was significantly higher than that in adopted rabbits (1.1%) (*p* value = 3.992 × 10^−5^) (Table 1). The prevalence of dermatophyte infection in rabbits which were reared with other rabbits in totals of three or more (37.5%) was significantly higher than that of those reared in totals of two rabbits (12.5%) or reared alone (9.4%) (*p* value = 0.007) (Table 1). In addition, the rabbits reared with dogs or cats (21.0%) had a significantly higher risk to have dermatophyte infection than those reared without other animals (8.8%) (*p* value = 0.017) (Table 1). The rabbits infested with fur mites (35.3%) hada significantly higher risk to have dermatophyte infection than those without ectoparasite infestation (10.0%) (*p* value = 0.012) (Table 1).

### 3.4. Dermatophyte Isolates and Pet Rabbit Characteristics

Twenty-nine dermatophyte isolates from recruited pet rabbits and rabbit characteristics are shown in Table 2. *T. mentagrophytes* complex isolates were mainly from mixed breed rabbits bought from pet shops. Among those 28 rabbits, 15 rabbits were reared in the cages, while 11 rabbits were reared indoors and the other 2 were reared both indoors and in cages. In addition, six rabbits that detected positive for the *T. mentagrophytes* complex were infested by fur mites (Table 2). *M. canis* was isolated from one male dodge rabbit bought from a pet shop. The rabbit was over 6 years old and reared in cage with another rabbit, in addition to having other animals in the house (Table 2). Only the rabbit infected with *M. canis* showed clinical signs of slight scratching and skin lesions, including alopecia and scurf.

### 3.5. Fitting Firth’s Bias Reduction Logistic Regression Model

Among the 12 categorical variables, source, number of rearing rabbits, rearing with other animals, and ectoparasite infestation were considered as significant factors associated with dermatophyte infection in pet rabbits. Because no dermatophyte infection was detected from pet rabbits sourced from personal breeding, the variable “source” was hypothesized as a virtual variable. Firth’s bias reduction logistic regression model was then created, where variables were selected using the forward approach, and only variables significantly contributing to the model were retained (R package logistf: Firth’s bias-reduced logistic regression version 1.21). The regression model is

Logistic (Y) = −1.701 − 2.747X_1_ − 2.816X_2_ + 0.221X_3_ + 2.435X_4_

where Y: whether the rabbit has dermatophyte infection, X_1_: whether the source of the rabbit is adoption, X_2_: whether the source of the rabbit is personal breeding, X_3_: whether the number of rearing rabbits is two, X_4_: whether the number of rearing rabbits is three or more than three (Table 3).

The interpretation of the variables obtained from the Firth’s bias reduction logistic regression model is mentioned as follows: the risk of dermatophyte infection in pet rabbits purchased from pet stores was 15.6 and 16.6 times higher than the rabbits from adoption and personal breeding, respectively; the risk of dermatophyte infection in pet rabbits reared with two rabbits was 1.247 times higher than the rabbits that were reared alone; the risk of dermatophyte infection in pet rabbits reared with three rabbits or more was 11.416 times higher than the rabbits that were reared alone.

## 4. Discussion

This is the first study on the prevalence of dermatophyte infection in pet rabbits in Taiwan and also the first report on the related risk factors in Asia. The prevalence of dermatophyte infection in pet rabbits (13.9%) in the present study was higher than those detected from veterinary clinics, pet shops, and pet cafés reported in Southern Italy (3.29%), the Netherlands (3.80%), Chile (7.14%), and Thailand (12.1%) [11,42,43,44]. By contrast, the prevalence was lower than those detected from veterinary clinics reported in Northern Italy (27.78%) and Bangladesh (88.89%) [7,12].

*T. mentagrophytes* is the most common dermatophyte species to infect rabbits from laboratories, veterinary clinics, pet shops, and rabbit farms [3,7,9,11,12,17,43,44,45]. In addition to *T. mentagrophytes*, the rabbits reared in the farms were also reported to be infected with *M. canis* [3,8,9,17]. Based on our findings, the *T. mentagrophyte* complex is also the predominant dermatophyte (96.5%) that infects pet rabbits in Northern Taiwan. After fungal infection was identified in pet rabbits, the treatment of Itraconazole (5 mg/kg) was given for at least 6 weeks. After 6 weeks of oral medication, dermatophyte culture from dander samples was performed. After the negative results were confirmed, Itraconazole (5 mg/kg) was applied for another 2 weeks.

In the present study, the risk of dermatophyte infection in pet rabbits purchased from pet stores (18.3%, 28/153) was 15.6 and 16.6 times higher than the rabbits from adoption (1.1%) and personal breeding (0%), respectively. The rabbits in pet shops were mainly brought from different commercial farms and reared until sold. Because dermatophyte spores can remain viable for many years, the contaminated cages or environment in pet shops can facilitate mutual infection among the rabbits [36,46]. In addition to exposure to a contaminated environment, the other factors, such as direct contact with infected animals, fomite transmission, grooming appliances, bedding, collars, and ectoparasites, can also contribute to the optimal conditions for dermatophyte infection [47,48]. Only one case of the dermatophyte infected rabbits in this study was an adopted individual (1.1%; 1/85). The adopted rabbits were mostly from the Taiwan Rabbit Saving Association, in which the rabbits came to the association from many different places for various reasons. The unclear history and improper living conditions could be related to their dermatophyte infection. The personal breeding rabbits were from friends or other families. These rabbits were usually taken care of and possibly given medicine for parasite prevention. Generally, two doses of Selamectin (18–20 mg/kg) with a 4-week interval were applied to rabbits by veterinarians every 6 months. Ectoparasite can disrupt the stratum corneum and cause itching, microtrauma, and scale production of the rabbit skins, and the injured skin can be the infected areas of arthrospores [48]. For this reason, the parasite prevention could reduce the risk of dermatophyte infection in rabbits.

Another risk factor of rabbit dermatophytosis in this study is the number of rearing rabbits. It was found that the more rabbits were reared together, the higher possibility of dermatophyte infection. *T. mentagrophytes* spores can not only be isolated from infected rabbits and asymptomatic carriers, but also from rabbit nests, cages, and the surrounding environment [3,4,49]. A previous study also reported that dermatophytes can be isolated from the two healthy rabbits that shared a cage with a third rabbit, which showed skin lesions because of dermatophyte infection [50]. 

*M. canis* had been predominantly reported on dogs and cats and has also been found on pet rabbits [18,51,52]. With the molecular evidence, the isolation of *M. canis* from rabbit samples could be due to the presence of dogs and cats in the surrounding environment, which can be an asymptomatic carrier for this dermatophyte species [10,51]. This possibility is also supported by our results, in that *M. canis* was isolated from the rabbit reared with dogs. On the other hand, *T. mentagrophytes* spores were detected from a dog’s basket, which showed the potentiality of dermatophyte infection in humans and other animals by direct contact with a dog or its living area [4]. 

In the present study, fur mite infestation was found to be associated with the dermatophyte infection. *Cheyletiella parasitovorax* is a non-burrowing fur mite, which is commonly found on rabbits and is transmitted among rabbits by direct contact. The obligate parasite causes a disruption of the stratum corneum, which may facilitate a dermatophyte infection during an infestation of rabbits [37]. Another study regarding dermatophyte detection in nine rabbits that visited the veterinary hospital reported that deworming status was not associated with dermatophyte infection in those rabbits; however, more relevant research data are still needed because of the small sample size [12]. 

Autumn was the period with the highest risk for dermatophyte infection in pets (including rabbits) in Italy [7]. However, no seasonal differences in the prevalence were found in this study. It was reported that increased temperature (>20 °C) and relative humidity between 62–65% can raise the risk of dermatophyte infection in rabbits [8]. Taiwan belongs to the sub-tropical and tropical regions; therefore, the hot and humid climate makes a suitable environmental condition for dermatophyte infection and transmission, and less seasonal variation on the prevalence of dermatophyte infection. In the present study, no gender-related differences in the prevalence were found, which is similar to the previous studies [7,12,17,20,45,53]. Although the findings of this study showed that the age ranges were not associated with dermatophyte infection in rabbits, age has been identified as a predisposing factor for dermatophytosis in rabbits, and young rabbits or immuno-compromised rabbits are considered to be the most susceptible to infection [11,17,45,50]. One possible reason is that the immune system has not completely developed, and there are lower levels of fungistatic fatty acids in the sebum of young rabbits [54,55].

The risk of human dermatophyte infection, especially *T. mentagrophytes*, was associated with dermatophytosis in rabbits which were reared in human living and working areas [4,9,14]. During this study, two veterinary colleagues were infected with dermatophytes, and both of them had skin lesions on their forearms. The affected skin areas appeared as rounded erythematous plaques combined with itching. Tiny particle protrusions were observed around the areas, and there was a clear scaly center with outward expansion, redness, and blisters at the edges. After applying antifungal ointment, the skin lesion was healed after two weeks. Because veterinary colleagues usually need to restrain rabbits on the table or hold rabbits in their arms while applying medicine, this is the possible reason for their dermatophyte infection. In a previous case report in Taiwan, a rabbit owner who had been diagnosed with dermatophytosis was infected with *T. benhamiae* and had a contact history with her two pet rabbits [56]. Furthermore, more than half of human cases of *T. benhamiae* infection had a history of contact with rabbits [57]. The common infected areas of rabbit owners include forearm, neck, body, face, head, and waist. The owners often held the rabbits in their arms, rubbed the rabbits on their faces, and/or put the rabbits on their abdomens to play when they lay on their beds, which increased the risk of dermatophyte infection in the rabbit owners.

Rabbits infected with dermatophytes in pet shops, farms, or houses are undesired. This is not only because of zoonotic exposure for humans, but also for the risk of spreading the infection to other animals. Pet shops are the main source of pet rabbits in Taiwan, and the rabbits from there have a higher risk for dermatophyte infection; therefore, preventive measures in pet shops and their source, rabbit farms, should be taken. The rabbits arriving in pet shops should be quarantined in a clean environment, and fungal culturing is an option to achieve dermatophyte-free animals [58]. In addition, placing more than one rabbit in the same cage should be avoided. Cages need to be regularly disinfected after cleaning, and exchanging bowls, food cups, or other material between cages needs to be prevented. For human infection, regular hygiene measures should be implemented after handling or working with animals, such as hand washing and changing clothes.

## Figures and Tables

**Table 1:** Numbers of positive pet rabbits for dermatophyte culture and PCR assay between different variables.

Variables	No. of Positive Rabbits/No. of Rabbits Tested (%)				*p* Value
Breed	Dodge	Dutch Dwarf	Mixed	Others	
	7/43 (16.3)	6/33 (18.0)	16/97 (16.5)	0/77 (0)	0.719
Age	<6 months	6 months to 6 years	>6 years		
	4/35 (11.4)	23/178 (12.9)	2/37 (5.4)		0.495
Gender	male	female			
	17/132 (12.9)	12/118 (10.2)			0.557
Neuter status	neutered	non-neutered			
	11/104 (10.5)	18/146 (12.3)			0.695
Source	pet shops	adoption	personal breeding		
	28/153 (18.3) ^a^	1/85 (1.1) ^b^	0/12 (0) ^b^		3.992 × 10^−5^
Living space	indoor	cage	indoor and cage		
	11/86 (12.8)	16/122 (13.1)	2/42 (4.7)		0.321
No. of rearing rabbits	one	two	three or more		
	19/202 (9.4) ^c^	4/32 (12.5) ^c^	6/16 (37.5) ^d^		0.007
Rearing with other animals	only rabbit	with dogs or cats			
	17/193 (8.8)	12/57 (21.0)			0.017
Ectoparasite infestation	fur mites	lice	non-parasite		
	6/17 (35.3) ^e^	0/3 (0) ^ef^	23/230 (10.0) ^f^		0.012
Seasons of sample collection	Spring (February to April)	Summer (May to July)	Autumn (August to October)	Winter (November to January)	
	8/73 (11.0)	10/63 (15.9)	7/68 (10.3)	4/46 (8.7)	0.692

Numbers with different superscripts differ significantly (*p* < 0.05) for No. of positive rabbits/No. of rabbit tested (%) in each variable.

**Table 2:** Dermatophyte isolates (*n* = 29) from recruited pet rabbits and their characteristics.

Variables	*T. mentagrophytes* Complex (*n* = 28)			*M. canis* (*n* = 1)
Breed	Dodge (*n* = 6)	Dutch Dwarf (*n* = 6)	Mixed Breed (*n* = 16)	Dodge
Age and gender	<6 months (1 M), 6 months to 6 years (3 M, 1 F), >6 years (1 M)	<6 months (1 F), 6 months to 6 years (3 M, 2 F)	<6 months (2 F), 6 months to 6 years (4 M, 9 F), >6 years (1 M)	>6 yrars (M)
Source	pet shop (*n* = 27), adoption (*n* = 1)			pet shop
Living space	cage (*n* = 15), indoor (*n* = 11), indoor and cage (*n* = 2)			cage
No. of rearing rabbits	1 rabbit (*n* = 19), 2 rabbits (*n* = 3), 3 rabbits or more (*n* = 6)			2 rabbits
Rearing with other animals	only rabbit (*n* = 16), dogs/cats (*n* = 12)			dogs/cats
Ectoparasite infestation	fur mites (*n* = 6), non-parasite (*n* = 22)			non-parasite

M, male; F, female.

**Table 3:** Firth’s bias reduction logistic regression in measuring variables related to dermatophyte infection in pet rabbits.

	Regression Coefficient	Standard Deviation	Chi-Square Value	*p* Value
intercept	−1.701	0.248	65.231	<0.001
X_1_	−2.747	0.879	19.532	<0.001
X_2_	2.816	1.668	5.768	0.016
X_3_	0.221	0.581	0.142	0.706
X_4_	2.435	0.711	12.218	<0.001

## Data Availability

Not applicable.
